# Relationship between aortic stiffness and modes of left ventricular deformation at rest and dobutamine-stress differ in the presence of preserved and compromised contractile capacity

**DOI:** 10.1186/1532-429X-13-S1-P130

**Published:** 2011-02-02

**Authors:** Valentina O Puntmann, Alun D Hughes, Rolf Gebker, Cosima Jahnke, Jesus G Mirelis, Bernhard Schnackenburg, Eckart Fleck, Frank Rademakers, Ingo Paetsch

**Affiliations:** 1 Imperial College London/The German Heart Institute Berlin, London, UK; 2 Imperial College London, London, UK; 3 The German Heart Institute Berlin, Berlin, Germany; 4 Catholic University Leuven, Leuven, Germany

## Objective

To investigate the phenotypic relationships between aortic stiffness and left ventricular (LV) stroke volume (SV) and modes of deformation (longitudinal - LD and radial -RD) at rest and stress, with preserved and compromised contractile capacity.

## Background

Increased aortic stiffness, measured by pulse wave velocity (PWV), is linked to a range of cardiovascular (CV) risk factors and acts as predictor of CV events, independently of age-related increase in pulse pressure. As an integrated marker of the pulsatile component of LV afterload, PWV has been linked to prognostically adverse cardiac phenotypes, including LV systolic dysfunction and post-infarct remodelling. Evidence suggests that longitudinal mode of deformation is particularly important to overcome the superimposed afterload. Thus, reduced LD due to myocardial ischaemia or scar may influence the capacity to offset the LV afterload in patients with increased aortic stiffness.

## Methods

**Figure 1:**
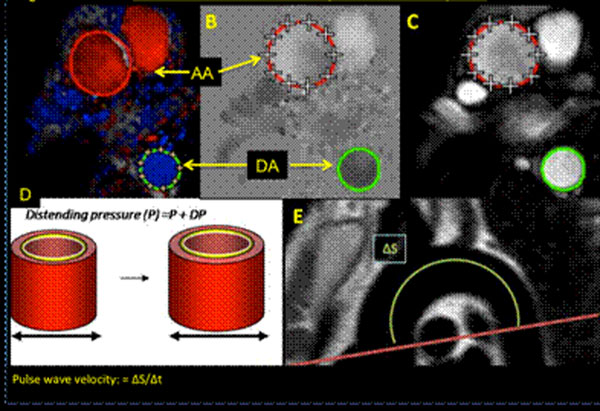
Aortic stiffness and distensibility measurements by MRI

At total of 124 cardiac patients underwent a high-dose dobutamine-stress/perfusion magnetic resonance (DS(P)MR) protocol with simultaneous late gadolinium enhancement (LGE) imaging and aortic pulse wave velocity (PWV) measurements (Figure 1).

## Results

**Figure 2:**
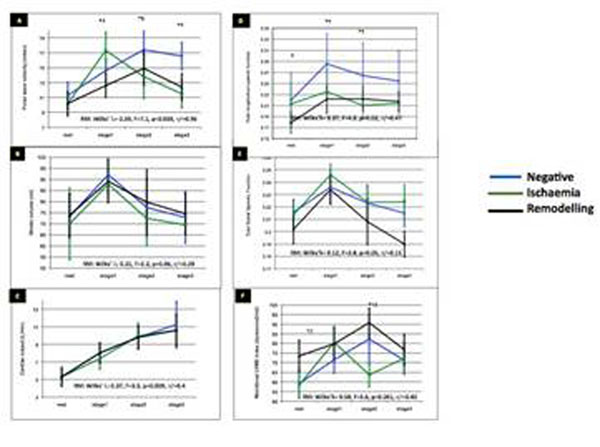
Measures at rest and dobutamine stress

Groups were classified according to the DS(P)MR/LGE outcome as negative (n=68) or positive for ischaemia (n=25), or post-infarct LV remodelling with scarring (n=31). All three groups showed a significant within-subject increase in HR and cardiac output with similar HR*BP products (p>0.05) in all 4 stages of the protocol (Figure 2A-F). SV increased at 20 mcg/kg/min (rest vs. stress20: SV: F=2.2, p=0.06) with a gradual reduction thereafter, associated with a step-wise decrease in cavity volumes. Changes in SV were paralleled by an increase in LD (rest vs. stress20: LD: F=4.9, p=0.02), more prominent in the negative group (30% vs. 10%, vs. 24%, F=7.7, p=0.001). Ischaemic and remodelled groups showed a late-stage increase and reduction of LV wall stress and RD, respectively. At rest, LD was the sole predictor of PWV in all groups at rest, and also with dobutamine-stress for the negative group, with late reversal into a positive relationship. In other groups, the association between PWV and total LD was negative throughout, with a differential relationship with total RD: positive in ischaemic and negative in remodelling.

## Conclusions

Our findings reveal that modes of deformation to increased aortic stiffness differ at rest and dobutamine-stress and with compromised LV contractility: intact myocardium relies on longitudinal function to displace volume and offset the afterload, whereas with LV dysfunction, increased LV wall stress reveals a role for radial component.

